# Experience with the Pascal^®^ photocoagulator: An analysis of over 1200 laser procedures with regard to parameter refinement

**DOI:** 10.4103/0301-4738.77007

**Published:** 2011

**Authors:** Saumil Sheth, Paolo Lanzetta, Daniele Veritti, Ilaria Zucchiatti, Carola Savorgnani, Francesco Bandello

**Affiliations:** Department of Ophthalmology, University of Udine, Udine, Italy

**Keywords:** Barrage photocoagulation, focal photocoagulation, laser power, laser spot size, laser treatment duration, panretinal photocoagulation, pascal^®^ photocoagulation

## Abstract

**Aim:**

To systematically refine and recommend parameter settings of spot size, power, and treatment duration using the Pascal^®^ photocoagulator, a multi-spot, semi-automated, short-duration laser system.

**Materials and Methods:**

A retrospective consecutive series with 752 Caucasian eyes and 1242 laser procedures over two years were grouped into, (1) 374 macular focal / grid photocoagulation (FP), (2), 666 panretinal photocoagulation (PRP), and (3) 202 barrage photocoagulation (BP). Parameters for power, duration, spot number, and spot size were recorded for every group.

**Results:**

Power parameters for all groups showed a non-gaussian distribution; FP group, median 190 mW, range 100 – 950 mW, and PRP group, median 800 mW, range 100 – 2000 mW. On subgroup comparison, for similar spot size, as treatment duration decreased, the power required increased, albeit in a much lesser proportion than that given by energy = power × time. Most frequently used patterns were single spot (89% of cases) in FP, 5 × 5 box (72%) in PRP, and 2 × 2 box (78%) in BP. Spot diameters as high as ≈ 700 μm on retina were given in the PRP group. Single session PRP was attempted in six eyes with a median spot count of 3500.

**Conclusion:**

Overall, due to the small duration of its pulse, the Pascal^®^ photocoagulator tends to use higher powers, although much lower cumulative energies, than those used in a conventional laser. The consequent lesser heat dissipation, especially lateral, can allow one to use relatively larger spot sizes and give more closely spaced burns, without incurring significant side effects.

The Pascal^®^ (Pattern Scan Laser) photocoagulator is a 532 nm, frequency-doubled, neodymium-doped, yttrium aluminum garnet (Nd:YAG), solid-state laser that can deliver multiple laser spots in a predetermined pattern array. If this has to be achieved in a semiautomated manner, that is, with a single foot pedal depression, albeit without the use of an expensive retinal tracker[1,2] or a cumbersome beam splitter (Bahmanyar S, Jones MS, Multi-spot laser surgery, USA patent 5,921,981 7/13/1999), the treatment needs to be given in successive spots in about the same time as that taken to deliver a single spot by conventional laser (100 – 200 ms).[3–5] Thus, for a 2 × 2 pattern array, approximately 20 ms (10 ms – 30 ms) should be the ideal treatment duration setting, allowing some time for the beam to move in between the spots. Shortening of this duration will, in turn, bring about alteration in other parameters, principally the power, if similar energy or end point burns have to be delivered on the retina. Of late, a retrospective analysis of 75 laser procedures using the Pascal^®^ photocoagulator[6] concluded that despite the shorter duration necessitating higher power use, the Pascal^®^ system is not only safe and effective, but may even offer potential advantages over the conventional laser. The aim of our study was to systematically identify and refine parameter settings of spot size, power, and treatment duration from the experience gained through the performance of 1242 laser procedures over two years, using various practical permutations and combinations.

## Materials and Methods

A retrospective study of a total of 752 Caucasian eyes or 1242 consecutive laser procedures was carried out at a single center (Department of Ophthalmology, University of Udine, Udine, Italy) employing a single Pascal^®^ laser system, from December 2006 to November 2008. A total of 1242 procedures were grouped as follows: (1) Focal and / or grid photocoagulation, (FP, n = 374 procedures) for diabetic macular edema, (2) Panretinal photocoagulation for proliferative diabetic retinopathy (PRP, n = 666 procedures), and (3) Barrage photocoagulation for retinal tears and holes (BP, n = 202 procedures).

All laser treatments were performed under topical anesthesia. Pupillary dilatation was achieved with 1% tropicamide and 10% phenylephrine eye drops, put once or twice, at ten minute intervals, half an hour before the procedure. For every patient, the best-corrected visual acuity (BCVA) was recorded before and after laser photocoagulation. The end point for FP was ‘just a visible’ burn (Grade I to II), while the end point for PRP and BP was a ‘clearly visible’ gray burn, not amounting to charring of the retinal tissue (Grade II to III).[7]

Principally, two different sets of parameters based on the laser spot size, treatment duration, and the type of lens used, were employed for each group of laser treatments, by two retinologists having sufficient experience (at least 10 years) in the use of retinal lasers, based solely upon their personal preferences and comfort levels. These parameters were later compared. Hence, FP was further subgrouped into, (a) 100 μm on the Volk^®^ quadraspheric lens with 20 ms pulse (FP100Q20, n = 188 treatments) and (b) 200 μm on the Goldmann three mirror lens with 30 ms pulse (FP200G30, n = 186 treatments). Similarly, PRP was further subgrouped into (a) 200 μm on the Volk^®^ quadraspheric lens with 20 ms pulse (PRP200Q20, n = 349 treatments) and (b) 400 μm on the Volk quadraspheric lens with 30 ms pulse (PRP400Q30, n = 317 treatments). When a pattern array was used, the spot separation was set at 0.5 times the burn width. The numbers of spots were recorded for each group of laser treatment along with the pattern of treatment on the Pascal system as a single spot, 2 × 2 grid, 3 × 3 grid, 5 × 5 grid, and so on. Although the pattern selected depended mainly upon the ease of its application, the final discretion was of the treating retinologist.

In spite of the large sample sizes, the gaussianity of the power parameters in all the three groups was tested by the D’Agustino-Pearson test, and while making comparisons between powers, non-parametric (Mann Whitney U test for independent samples) in preference to parametric (unpaired t test with unequal variances) statistical tests were utilized, wherever appropriate. The level of significance was set at *P* < 0.05. Data were analyzed using SPSS (Statistical Package for Social Sciences, version 14).

## Results

The power parameters in all the three groups showed a non Gaussian distribution [Table 1]. The power parameters overall in the FP group were as shown in Table 1, with a range from 100 mW to 950 mW and median 190 mW. In the FP100Q20 subgroup, the power parameters were as shown in Table 2, with a range from 100 mW to 950 mW and median 187.5 mW. In the FP200G30 group, the power parameters were as shown in Table 2, with a range from 100 mW to 950 mW and median 200 mW. There was no significant difference noted in the power parameters between the two subgroups on non-parametric (*P* = 0.22) testing. When the energy parameters (energy = power × time) were compared between the two subgroups, there was a very significant difference noted by non-parametric (*P* < 0.001) testing.

**Table 1:** Dispersion plots of the powers of the focal photocoagulation, panretinal photocoagulation, and barrage photocoagulation groups showing non-Gaussianity of distribution

Group	FP (n = 374)	PRP (n = 666)	BP (n = 202)
Range (mW)	100 – 950	100 – 2000	125 – 1800
Mean ± SD (mW)	215.9 ± 109.9	876.1 ± 362.7	500.7 ± 264.9
95% CI for the mean	204.8 to 227.1	848.5 to 903.7	463.8 to 537.6
Median (mW)	190	800	450
95% CI for the median	175 to 200	775 to 800	400 to 495
Coefficient of Skewness	2.4 (*P* < 0.0001)	0.98 (*P* < 0.0001)	1.83 (*P* < 0.0001)
Coefficient of Kurtosis	10.1 (*P* < 0.0001)	0.38 (*P* = 0.0700)	4.96 (*P* < 0.0001)
D’Agostino-Pearson test for Normal distribution	Reject normality (*P* < 0.0001)	Reject normality (*P* < 0.0001)	Reject normality (*P* < 0.0001)

SD: Standard deviation, FP: Focal photocoagulation, PRP: Panretinal photocoagulation, BP: Barrage photocoagulation

**Table 2:** Comparison of powers and energies between the Focal photocoagulation subgroup with 100 μ spot size setting using the Volk^®^ quadraspheric lens with 20 ms treatment duration (FP100Q20) and the Focal photocoagulation subgroup with a 200 µ spot size setting, using Goldmann three mirror lens with 30 ms treatment duration (FP200G30), showing a nonsignificant difference in powers, but a very significant difference in energies

	FP100Q20 (n = 188)	FP200G30 (n = 186)
Power		
Mean (mW) ± SD	212.1 ± 110.7	219.8 ± 109.2
95% CI for the mean	196.2 to 228.0	204 to 235.6
*P* value (t test)		*P* = 0.49
Range (mW)	100 – 950	100 – 925
Median	187.5	200
95% CI for the median	175 to 200	200 to 200
*P* value (Mann Whitney U test)		*P* = 0.23
Energy		
Mean ± SD (mW)	4241.7 ± 2214.2	6595.2 ± 3275.5
95% CI for the mean	3923.1 to 4560.3	6121.3 to 7068.9
*P* value (t test)		*P* < 0.001
Range (mJ)	2000 – 19000	3000 – 27750
Median (mJ)	3750	6000
95% CI for the median	3500 to 4000	6000 to 6000
*P* value (Mann Whitney U test)		*P* < 0.0001

SD = Standard deviation

The power parameters overall in the PRP group were as shown in Table 1, with range from 400 mW to 2000 mW and median, 800 mW. In the PRP200Q20 subgroup, the power parameters were as shown in Table 3, with a range from 400 mW to 1850 mW and median, 700 mW. In the PRP400Q30 subgroup, the power parameters were as shown in Table 3, with a range from 400 mW to 2000 mW and median, 900 mW (note that the power parameters were taken of spots around the vascular arcade). There was a significant difference noted in the power parameters between the subgroups by non-parametric (*P* < 0.001) testing. When the energy parameters (energy = power × time) were compared between the two subgroups, there was a very significant difference noted on non-parametric (*P* < 0.001) testing.

**Table 3:** Comparison of powers and energies between the Panretinal photocoagulation subgroup with a 200 μ spot size setting using the Volk^®^ quadraspheric lens with 20 ms treatment duration (PRP200Q20) and Panretinal photocoagulation subgroup with a 400 μ spot size setting using the Volk^®^ quadraspheric lens with 30 ms treatment duration (PRP400Q30) showing a very significant difference in powers and energies

	PRP200Q20 (n = 349)	PRP400Q30 (n = 317)
Power		
Mean ± SD (mW)	794.3 ± 334.9	967 ± 369.4
95% CI for the mean	759 to 829.5	926.2 to 1007.9
*P* value (t test)		*P* < 0.0001
Range (mW)	400 – 1850	400 – 2000
Median (mW)	700	900
95% CI for the median	625 to 750	850 to 975
*P* value (Mann Whitney U test)		*P* < 0.0001
Energy		
Mean ± SD (mJ)	15885.7 ± 6697.8	29011 ± 11082.9
95% CI for the mean	15180.6 to 16590.9	27786.3 to 30235.8
*P* value (t test)		*P* < 0.0001
Range (mJ)	8000 – 37000	12000 – 60000
Median (mJ)	14000	27000
95% CI for the median	12500 to 15000	25500 to 29250
*P* value (Mann Whitney U test)		*P* < 0.0001

SD = Standard deviation

Moreover, in the PRP200Q20 subgroup where 105 eyes underwent three complete sessions of PRP, the spot number required ranged from 965 to 4304, with 1390 being the median. In the PRP400Q30 subgroup where 84 eyes underwent three complete sessions of PRP, the number of spots ranged from 846 to 2333 with 1203 being the median. There were a significantly greater number of spots required in the PRP200Q20 subgroup than in the PRP400Q30 subgroup by non-parametric (*P* < 0.001) testing.

Coming to the BP group, the overall power parameters were as shown in Table 1, with a range from 125 mW to 1800 mW and median, 450 mW for a spot size of either 200 μm with 20 ms duration or 400 μm with 30 ms duration.

Considering the fact that the ophthalmoscopically visible burn intensity was similar in the PRP and BP groups, and the fact that the BP group burns were given in the mid and the far periphery in all the cases, the power parameters of the BP group burns should be in effect, similar to those of the PRP group burns, when given in the same area. When this extrapolation was applied, the energy (power × time) parameters for photocoagulation used in the mid or far periphery significantly reduced compared to those used in the region of the vascular arcades or posterior pole on non-parametric (*P* < 0.001) testing [Table 4]. It is to be noted that the combined data for the PRP200Q20 and PRP400Q30 subgroups was used when comparing against BP and that the proportion of 200 to 400 μm spot sizes used in the PRP (349/666, 52.4%) and BP (104/202, 51.49%) groups were matched for significance by the Fischer’s exact test (*P* = 1.0).

**Table 4:** Comparison of energies between central and peripheral photocoagulation showing a very significant difference in energies

Energy	Central photocoagulation (n = 666)	Peripheral photocoagulation (n = 202)
Mean ± SD (mJ)	22133.1 ± 11174.9	11967.7 ± 7911.2
95% CI for the mean	21282.8 to 22983.3	10867.3 to 13067.9
*P* value (t test)		*P* < 0.001
Range (mJ)	8000 – 60000	2500 – 54000
Median (mJ)	19000	10000
95% CI for the median	18000 to 20250	9000 to 10500
*P* value (Mann Whitney U test)		*P* < 0.0001
		

SD = Standard deviation

The most frequently used patterns were single spot (89% of cases) in FP, 5 × 5 box (72%) in PRP, and 2 × 2 box (78%) in BP. No complications were encountered in the FP and BP groups. In the PRP group, three cases of retinal haemorrhage, secondary to a micro-explosion, and two cases of choroidal detachment were reported. However, these cases self-resolved over time without any significant intervention. A single session PRP was attempted in six eyes. The median number of spots given in these six cases with a 200 μm spot size and duration of 20 ms was 3500.

## Discussion

When carrying out a descriptive analysis, we also attempted to elucidate the relationship between the three principle Pascal^®^ laser parameters of power (P), time (t), and area (A) that govern the overall energy (E) delivery to the retina. This is given by the formula: E = P × t / A, where A = πr^2^, r being the spot radius, which is half of the spot size.

In the Pascal^®^ FP group, combining the subgroups, our typical parameters were approximately: power, 200 – 250 mW, duration, 20 – 30 ms, spot size, and 150 – 200 μm on the retina (the subgroup using 100 μm spot on the Volk^®^ quadraspheric lens amounts to 150 – 175 μm on the retina), as against the typical parameters employed by the conventional laser for FP, as per the early treatment of diabetic retinopathy study (ETDRS),[4,5] that is, power, 100 mW, duration, 100 – 150 ms, and size, 100 – 150 μm on the retina. Similarly, in the Pascal^®^ PRP group, combining the subgroups, our typical parameters were approximately: power, 700 – 900 mW, duration, 20 – 30 ms, spot size, 200 – 400 μm (amounting to 350 – 700 μm using the Volk^®^ quadraspheric lens on the retina), as against the typical parameters employed by the conventional laser for PRP as per the ETDRS and the diabetic retinopathy study (DRS),[4–6] namely, power, 200 – 400 mW, duration, 150 – 250 ms, and spot size, 500 μm on the retina. For an equivalent spot size, the powers used in the Pascal^®^ FP and PRP groups doubled or tripled those used in the conventional laser FP or PRP groups.

This is hardly surprising, as shorter durations used in the Pascal^®^ groups (one-fifth of that used for FP and one-seventh of that used for PRP when compared with the conventional laser groups) can account for a greater use of power. Nevertheless, when applying E = P × t / A, for similar spot sizes, the cumulative energy used in the conventional laser groups (FP = 100 mW × 125 ms = 12500 mJ, PRP = 300 mW × 200 ms = 60000 mJ) far exceeds that used in the Pascal^®^ groups (FP = 250 mW × 25 ms = 6250 mJ, PRP = 800 mW × 25 ms = 20000 mJ). We have animal studies which show that for similar spot sizes, as the pulse duration decreases, the power does increase, although in a much lesser proportion.[8] Moreover, for increasing spot sizes, the power continues to increase in a fairly linear proportion, as against the duration, which increases exponentially.[9] This implies that if 6250 mJ (instead of 12500 mJ) or 20000 mJ (instead of 60000 mJ) is all the cumulative energy that may be required by the Pascal^®^ laser to reach similar end points (Grade I to II burns in the FP group and II to III burns in the PRP group), a whole lot of extra energy delivered by the conventional laser may be getting dissipated laterally or vertically, more so when large spot sizes are selected. Lateral dissipation may account for spot size enlargement (atrophic creep) over time.[10] This is potentially harmful, as spot coalescence, when not spaced adequately, may give rise to annoying scotomas. Vertical dissipation may result in full thickness retinal scarring,[9] with choroidal involvement, causing greater pain.[11] On the upside, this very heat dissipative nature of the burns delivered by conventional laser may help in achieving a fair margin between the lowest power producing a Grade I burn and the highest power causing a Bruch’s membrane rip or hemorrhage, especially when small spot sizes are selected. Studies have shown that despite using durations of 20 ms, this margin (lowest 110 mW to highest 600 mW) is still sufficiently wide to allow room for power errors,[8] even at spot sizes as low as 130 μm.[9] At durations of around 1 ms, this margin of error closes down to zero.[9]

Therefore, 20 ms, or for that matter 10 to 30 ms, seems to be the most appropriate spot duration. Lesser energy dissipation can amount to a greater predictability of spot size, even when retinal spot sizes as high as 700 μm are used by the PRP400Q30 subgroup (400 μm on the Volk^®^ quadraspheric lens amounts to 700 μm on the retina). The 500 μm retinal spot size routinely used for PRP by the conventional laser, with its much longer pulse duration of 200 ms, will eventually expand to 700 μm or perhaps, even greater, This situation causes greater harm, especially when the enlarging spot encroaches on the space between two spots and fills it up gradually, causing total coalescence. Thus, in the Pascal^®^ laser, instead of spreading burns one burn width apart, one can give a much tighter and uniform PRP with 0.5 burn width gap between the burns, without worrying much about spot coalescence.

There is no doubt that the number of spots required in the PRP200Q20 subgroup would be greater than those in the PRP400Q30 subgroup. However, the number of spots in the PRP400Q30 subgroup (≈ 1200) was found to be much lesser than those used in a conventional laser (≈ 2000).[3–5] This is probably because the PRP400Q30 subgroup can afford to use a greater spot diameter (> 700 μm on the retina) when compared to the conventional laser (500 μm on the retina), [3–5] despite an equivalent spot separation between them (0.5 burn widths with 400 μm is roughly equivalent to one burn width, with 200 – 300 μm). Not only does the Pascal^®^ laser enhance the speed of laser delivery (1 – 3 minutes per session instead of 10 – 15 minutes) on account of it giving multiple spots of short duration as shown by Blumenkranz *et al*,[8] but also, overall, a lesser number of spots may be required for a complete PRP. Recent reports have shown greater success in terms of neovascularization regression, with extensive treatment in the first session itself[12] or ablating larger areas of the retina, inclusive of its far periphery.[13] In both these cases, the Pascal^®^ laser could prove to be of a greater convenience than the conventional laser.

Coming to subgroup comparisons in the Pascal^®^ FP group, as pointed earlier, a 100 μm spot size in the FP100Q20 subgroup on the Volk^®^ quadraspheric lens amounts to 150 – 175 μm on the retina, somewhat equivalent to 200 μm employed in the FP200G30 subgroup. Thus, ideally, if the same end point (Grade I to II) burn has been used, the energies (P × t) in the subgroups should not differ. Our result does confirm similar powers, although with varying durations. This means that the FP200G30 subgroup is using significantly greater energy. It may, hence, be extrapolated that even duration pulses of 30 ms, as against 20 ms, do show significant lateral, if not vertical energy dissipation. Comparing our series with the one by Sanghvi *et al*,[6] where a smaller spot duration of 10 ms and still smaller spot size of 100 μm has been used for FP, the powers as well as the energies are significantly lower than those used in our series.

Although the two cases of choroidal detachments were self-resolving and single session PRPs seem to be a very feasible option now with the Pascal^®^ laser, with equivalent, if not better, long-term results in terms of regression of neovascularization,[14] we still recommend caution with spot counts going over 3000.

To conclude, our predominantly descriptive study of 1242 procedures may help the novice Pascal^®^ laser users to refine laser parameters and apply parameter algorithms that best suit their needs in terms of accuracy, safety, speed, and patient comfort.

## References

[1] Wright CH, Ferguson RD, Barrett SF, Rylander HG, Welch AJ, Oberg ED (1997). Hybrid retinal photocoagulation system using analog tracking. Biomed Sci Instrum.

[2] Oberg ED, Barrett SF, Wright CH (1997). Development of an integrated automated retinal surgical laser system. Biomed Sci Instrum.

[3] (1987). Techniques for scatter and local photocoagulation treatment of diabetic retinopathy: Early Treatment Diabetic Retinopathy Study Report no. 3. The Early Treatment Diabetic Retinopathy Study Research Group. Int Ophthalmol Clin.

[4] (1987). Treatment techniques and clinical guidelines for photocoagulation of diabetic macular edema: Early Treatment Diabetic Retinopathy Study report number 2. Ophthalmology.

[5] (1981). Photocoagulation treatment of proliferative diabetic retinopathy: Clinical application of Diabetic Retinopathy Study (DRS) findings, DRS report number 8. Ophthalmology.

[6] Sanghvi C, McLauchlan R, Delgado C, Young L, Charles SJ, Marcellino G (2008). Initial experience with the Pascal photocoagulator: A pilot study of 75 procedures. Br J Ophthalmol.

[7] Tso MO, Wallow IH, Elgin S (1977). Experimental photocoagulation of the human retina, correlation of physical, clinical and pathologic data. Arch Ophthalmol.

[8] Blumenkranz MS, Yellachich D, Andersen DE, Wiltberger MW, Mordaunt D, Marcellino GR (2006). Semiautomated patterned scanning laser for retinal photocoagulation. Retina.

[9] Jain A, Blumenkranz MS, Paulus Y, Wiltberger MW, Andersen DE, Huie P (2008). Effect of pulse duration on size and character of the lesion in retinal photocoagulation. Arch Ophthalmol.

[10] Morgan CM, Schatz H (1989). Atrophic creep of the retinal pigment epithelium after focal macular photocoagulation. Ophthalmology.

[11] Al-Hussainy S, Dodson PM, Gibson JM (2008). Pain response and follow-up of patients undergoing panretinal laser photocoagulation with reduced exposure times. Eye (Lond).

[12] Bailey CC, Sparrow JM, Grey RH, Cheng H (1999). The national diabetic retinopathy laser treatment audit. Eye (Lond).

[13] Cordeiro MF, Stanford MR, Phillips PM, Shilling JS (1997). Relationship of diabetic microvascular complications to outcome in panretinal photocoagulation treatment of proliferative diabetic retinopathy. Eye (Lond).

[14] Doft BH, Blankenship GW (1982). Single versus multiple treatment sessions of argon laser panretinal photocoagulation for proliferative diabetic retinopathy. Ophthalmology.

